# Food fussiness and food neophobia share a common etiology in early childhood

**DOI:** 10.1111/jcpp.12647

**Published:** 2016-10-14

**Authors:** Andrea D. Smith, Moritz Herle, Alison Fildes, Lucy Cooke, Silje Steinsbekk, Clare H. Llewellyn

**Affiliations:** ^1^ Department of Epidemiology and Public HealthHealth Behaviour Research CentreUniversity College LondonLondonUK; ^2^ Department of PsychologyNorwegian University of Science and Technology (NTNU)TrondheimNorway

**Keywords:** Eating behavior, twin design, behavioral genetics, food fussiness, food neophobia

## Abstract

**Background:**

‘Food fussiness’ (FF) is the tendency to be highly selective about which foods one is willing to eat, and emerges in early childhood; ‘food neophobia’ (FN) is a closely related characteristic but specifically refers to rejection of unfamiliar food. These behaviors are associated, but the extent to which their etiological architecture overlaps is unknown. The objective of this study was to quantify the relative contribution of genetic and environmental influences to variation in FF and FN in early childhood; and to establish the extent to which they share common genetic and environmental influences.

**Method:**

Participants were 1,921 families with 16‐month‐old twins from the Gemini birth cohort. Parents completed the Child Eating Behaviour Questionnaire which included three FF items and four FN items. Bivariate quantitative genetic modeling was used to quantify: (a) genetic and environmental contributions to variation in FF and FN; and (b) the extent to which genetic or environmental influences on FF and FN are shared across the traits.

**Results:**

Food fussiness and FN were strongly correlated (*r* = .72, *p* < .001). Proportions of variation in FF were equally explained by genetic (.46; 95% CI: 0.41–0.52) and shared environmental influences (.46; 95% CI: 0.41–0.51). Shared environmental effects accounted for a significantly lower proportion of variation in FN (.22; 95% CI: 0.14–0.30), but genetic influences were not significantly different from those on FF (.58, 95% CI: 0.50–0.67). FF and FN largely shared a common etiology, indicated by high genetic (.73; 95% CI: 0.67–0.78) and shared environmental correlations (.78; 95% CI: 0.69–0.86) across the two traits.

**Conclusions:**

Food fussiness and FN both show considerable heritability at 16 months but shared environmental factors, for example the home environment, influenced more interindividual differences in the expression of FF than in FN. FF and FN largely share a common etiology.

## Introduction

During early childhood, children are gradually introduced to an increasingly varied diet comprising previously unseen foods of different flavors, textures and visual characteristics. While some children willingly accept new foods, many are hesitant. These behaviors can be broadly characterized as food fussiness (FF) and food neophobia (FN), and are characteristics of early childhood eating problems seen in clinical settings (Bryant‐Waugh, Markham, Kreipe, & Walsh, 2010). FF is the tendency to be selective about the foods one is willing to try, often focusing on food‐specific attributes such as texture; FN is an overlapping construct but refers specifically to the refusal to try unfamiliar foods (Smith, Roux, Naidoo, & Venter, 2005). FN tendencies primarily exert themselves during the first tasting phase with a food item but FF persists beyond this initial encounter (Brown, 2010). Two studies found FF and FN to be associated, although the size of the correlation differed considerably (Galloway, Lee, & Birch, 2003: *r* = .19, *p* < .01; Finistrella et al., 2012: *r* = .53, *p* < .001). Parents consider FF and FN problematic because excessively fussy children may eat too little or a restricted number of foods; and excessive fussiness has been associated with failure to thrive (Wright & Birks, 2000).

Fussiness is also associated with behavioral problems (Jacobi, Agras, Bryson, & Hammer, 2003), and concurrent and prospective symptoms of anxiety and depression (Zucker et al., 2015). Interestingly, FN is associated with characteristics such as shyness or inhibition (Galloway et al., 2003); traits with established genetic influence. FF, however, has been linked more with environmental influences including breastfeeding duration or persuasive feeding practices (Carruth et al., 1998; Galloway et al., 2003). This raises the possibility that their etiology differ, suggesting that a child is more genetically predisposed to FN, but that experiential factors are crucial in expression of FF. If similar clinical presentations of feeding problems have different etiologies, identification of such differences may be vital to developing more efficient interventions (Kreipe & Palomaki, 2012). On the other hand, should FF and FN share their genetic and environmental etiology to a great extent, then these food avoidant traits do not have to be treated as distinct behaviors. A broader classification of feeding difficulties may therefore be of clinical use; common treatments would be justified if the genetic and environmental influences are largely the same. This would simplify treatment plans for healthcare professionals involved in the care of children with FF or FN.

Twin studies can be used to compare the relative contribution of genes and environment to variation across different traits. Two pediatric twin studies have reported substantial genetic contributions to FN, with heritability estimates of .78 in 4‐ to 7‐year olds (Cooke, Haworth, & Wardle, 2007) and .72 in 8‐ to 11‐year olds (Faith, Heo, Keller, & Pietrobelli, 2013). The only adult twin study found comparably high heritability (.69; Knaapila et al., 2011).

A direct comparison of the relative influence of genes versus environment on FF and FN in the same sample would help clarify the extent to which these traits share an etiology; an essential question in the understanding of food avoidance in children (Lafraire, Rioux, Giboreau, & Picard, 2015). We use data from a large pediatric twin study to estimate for the first time the genetic and environmental contributions to both FF and FN at 16 months of age, and the extent to which they share common genetic and environmental influences.

## Method

### Sample

Data were from Gemini, a prospective population‐based birth cohort of twins born in England and Wales in 2007. Two thousand four hundred and two families completed the baseline questionnaire. Gemini twins are representative of UK twins in terms of anthropometrics, sex, zygosity, and gestational age. As with many cohort studies, White‐British families and married couples are overrepresented; and parents report below average BMIs and healthier food habits than national averages (Van Jaarsveld, Johnson, Llewellyn, & Wardle, 2010).

### Zygosity and gestational age

Opposite‐sex twins were classified as dizygotic (DZ). A 20‐item questionnaire was used to determine the zygosity of same‐sex twins, which has been shown to be 95% accurate when validated against DNA markers (Price et al., 2012). Gestational age was parent‐reported as the number of weeks the mother had been pregnant at the time of delivery.

### Food fussiness and food neophobia

Parents completed the ‘food fussiness’ scale of the Child Eating Behaviour Questionnaire (CEBQ) for each twin when they were 16‐month old. The CEBQ has good internal and external reliability (Sleddens, Kremers, & Thijs, 2008; Wardle, Guthrie, Sanderson, & Rapoport, 2001), and the traits are stable over time (Ashcroft, Semmler, Carnell, van Jaarsveld, & Wardle, 2008). Before sending out the CEBQ to the Gemini families at 16 months, we piloted all items with a sample of 12 mothers with toddlers, recruited from children's centers in London. Mothers completed the CEBQ and took part in follow‐up interviews to establish if the items and response scale were age‐appropriate for 16‐month‐old children. Modifications were made to some scales, but the FF scale remained unchanged. The FF scale includes six items describing behaviors indicative of FF (two items) or FN (four items) on a 5‐point Likert scale (‘never’ to ‘always’). The two FF items ask about the variety of foods eaten (‘My child enjoys a wide variety of foods’), and how difficult it is to please the child with meals (‘My child is difficult to please with meals’). An additional FF item was added that asked about the child's refusal to eat certain types of foods (‘My child refuses certain types of food’). The four FN items assess the child's interest in tasting unfamiliar foods (‘My child refuses new foods at first’, ‘My child decides that s/he doesn't like a food, even without tasting it’, ‘My child is interested in tasting food s/he hasn't tasted before’ and ‘My child enjoys tasting new foods’). Participants had to have completed a minimum number of items (2/3 for FF, 3/4 items for FF) to be included in the analyses.

A principal components analysis established that the original factor structure of the CEBQ was replicated in the 16‐month‐old sample, and all of the FF items loaded onto one factor with high loadings (all ≤ .7). The FF and FN scales had good internal consistency (FF: Cronbach's *α* = .77; FN: Cronbach's *α* = .84). The FF and FN scales are shown in Table S1.

### Statistical analyses

Because twins share their age, and sex is correlated for same‐sex twins, it is standard practice to regress scores on gestational age, age at measurement, and sex, prior to heritability analyses to ensure these factors do not inflate the shared environmental effect. Pearson's correlation was used to establish the association between FF and FN. The twin design was used to establish the genetic and environmental influences on FF and FN.

#### Intraclass correlations

The relative importance of genes and environment on a given characteristic can be established by comparing the degree of resemblance between monozygotic (MZ) pairs (who share 100% of their genes) with that between DZ pairs (who share 50% of their segregating genes), using intraclass correlations (ICCs). Higher within‐pair resemblance for MZ than DZ pairs indicates a genetic contribution to variation in a trait. Cross‐twin cross‐trait (CT‐CT) correlations indicate the extent to which common genetic or environmental factors explain the phenotypic correlation between two traits. CT‐CT correlations related twin 1's FF to twin 2's FN, and vice versa. Similar patterns to the simple ICCs indicate the extent of common genetic or environmental contributions to the phenotypic association; for example higher CT‐CT correlations for MZ pairs than DZ pairs suggest that common genetic factors largely explain the phenotypic association. Simple ICCs for FF and FN, and CT‐CT ICCs were performed in SPSS Version 22 (IBM Corp, Armonk, NY).

#### Maximum likelihood structural equation modeling

Maximum likelihood structural equation modeling was used to derive reliable estimates of additive genetic (A), shared environmental (C), and unique environmental (E) effects, with 95% confidence intervals (95% CI), and provide goodness‐of‐fit statistics. Univariate models were used to estimate A, C, and E separately for FF and FN. This analysis permitted direct comparison of the relative importance of genes versus environment for FN and FF. A bivariate correlated factors model was used to estimate the extent of shared genetic and environmental influences underlying FF and FN, indicated by the etiological correlations. These establish the extent to which the genetic (*r*_A_), shared environmental (*r*_C_), and unique environmental influences (*r*_E_) are the same for both FF and FN. Etiological correlations are interpreted similarly to a Pearson's correlation coefficient, and range from −1 to 1; a high *positive r*_A_ indicates that most of the genetic influences on FF also influence FN; a low *positive r*_A_ indicates that few of the genetic influences are shared; a high *negative r*_A_ indicates that most of the genes that make an individual score high on FF, are the same as those that make them score low on FN.

The bivariate model also provides bivariate estimates of A, C, and E, which indicate the extent to which common genetic (bivariate A), shared (bivariate C), and unique environmental (bivariate E) influences drive the phenotypic association between two traits – that is the relative importance of shared genetic or environmental factors in driving the phenotypic association between FF and FN. The bivariate estimates are reported as proportions of the phenotypic association, and add up to 1.

Maximum likelihood structural equation modeling was performed in R (R Core Team, 2015), using OpenMx, version 2.2.6 (Boker et al., 2011). The Bayesian information criterion (BIC) fit statistic was used because it takes account of the sample size and the number of parameters in the model (Posada & Buckley, 2004). Additionally, goodness‐of‐fit is indicated by the likelihood ratio test, a procedure used to select the best‐fitting model among hierarchical nested models. The model with the lowest BIC value and smallest Δ*χ* ^2^ was chosen as the best‐fitting model. For univariate and bivariate analyses, after fitting a saturated model with no parameter constraints (i.e. means, variances, and covariances only), an ACE model was fitted to the data and compared to the saturated model. Subsequent submodels were run, dropping A, C, or A and C, to see if a more parsimonious model could be fitted. To test for sex differences in the etiology of each of FF and FN, sex‐limitation models were run. Fit statistics indicated no sex differences for FN. There was some suggestion that estimates might vary in effect size (so‐called ‘quantitative’ sex differences) for FF. However, the estimates derived for FF and FN were not significantly different for males and females, because the 95% confidence intervals did not overlap (fit statistics and parameter estimates for the sex‐limitation models are presented in Tables S2 and S3). Therefore, a bivariate model that combined males and females was used.

## Results

### Sample characteristics

The sample analyzed in this study included 1,932 families with available data on FF and FN. The sample characteristics are shown in Table 1.

**Table 1:** Characteristics of the Gemini sample (*n* = 3,864 children)

Twin pairs	*N* (%) or mean (*SD*)
Total	1,932
Zygosity	
MZ	626 (32.4)
DZ	1,306 (67.6)
Sex	
Males	1,909 (49.4)
Females	1,955 (50.6)
Gestational age (weeks)	36.26 (2.43)
Weight at birth (kg)	2.46 (0.54)
Age at questionnaire completion (months)	15.82 (1.15)
Food fussiness	2.05 (0.73)
Food neophobia	2.31 (0.76)

DZ, dizygotic; MZ, monozygotic.

Food fussiness and FN were positively correlated, and the effect size was large (*r* = .72, *p* < .001), showing that individuals who were fussy tended also to be neophobic.

### Comparison of the magnitudes of genetic and environmental influences underlying FF and FN

#### Twin correlations

**Figure 1:**
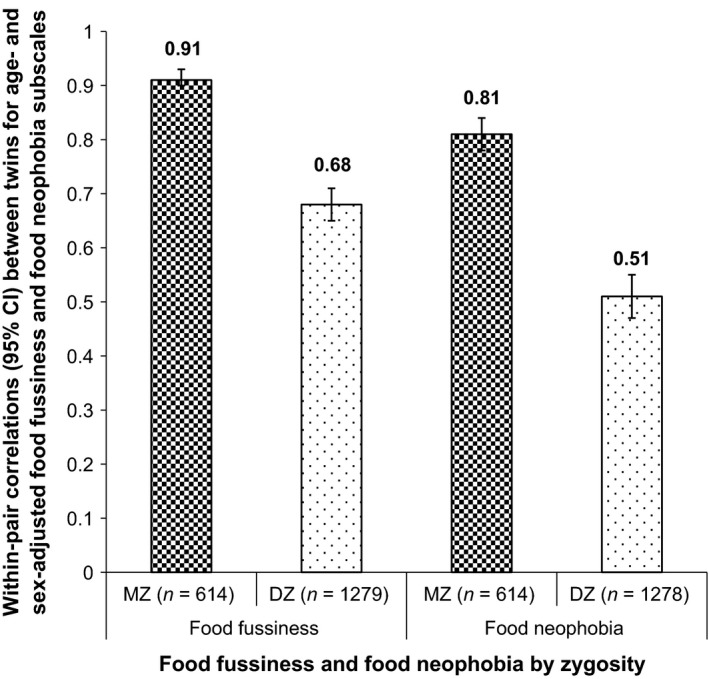
Intraclass correlations (ICCs) for food fussiness (FF) and food neophobia (FN) by zygosity. ICCs (95% CI) of FF and FN scores for MZ and DZ twin pairs to establish within‐pair similarity. FF and FN scores were standardized for gestational age, age at parental completion of the questionnaire and sex. MZ, monozygotic twins; DZ, dizygotic twins; *n*, number of twin pairs

The ICCs for MZs and DZs for FF and FN are shown in Figure 1. The ICCs for MZ twins were high and significant for both FF (.91; 95% CI: 0.90–0.93) and FN (.81; 95% CI: 0.78–0.84). The ICCs for the DZs were also significant, *and* were significantly lower than those for the MZs, because the 95% CIs of the estimates did not overlap those for the MZs for either trait (FF: .68; 95% CI: 0.65–0.71; FN: .51; 95% CI: 0.47–0.55). This pattern of resemblance indicates substantial genetic influence on both behaviors.

#### Maximum likelihood structural equation modeling

Parameter estimates and fit statistics for the full ACE model and submodels are shown in Table 2. In line with the pattern of resemblance for the ICCs, additive genetic effects were moderate for FF (.46; 95% CI: 0.41–0.52) and FN (.58; 95% CI: 0.50–0.67). The magnitude of the genetic effect was not significantly different, shown by the overlapping 95% confidence intervals. However, the effect of the shared environment was significantly lower for FN (.22; 95% CI: 0.14–0.30) than for FF (.46; 95% CI: 0.40–0.51); and the nonshared environment (E) explained significantly more of the variance in FN (.19; 95% CI: 0.17–0.22) than in FF (.09; 95% CI: 0.08–0.10).

**Table 2:** Model fit and parameter estimates for the saturated, ACE model, and submodels for food fussiness (FF) and food neophobia (FN)^a^

	Additive genetic effect (A)	Shared environment effect (C)	Nonshared environment effect (E)^b^	−2LL^c^	*df* c	BIC^c^	Δ BIC	Δ −2LL (*df*)	*p*‐Value
FF 16 months									
Saturated				8799.936	3776	−9846.725			
ACE	.46 (0.41–0.52)	.46 (0.40–0.51)	.09 (0.08–0.10)	8812.198	3781	−9859.459	−12.734	12.262 (5)	0.03
CE^d^	–	.76 (0.74–0.78)	.24 (0.22–0.26)	9100.119	3782	−9719.272	140.187	287.921 (1)	0.00
AE^d^	.91 (0.90–0.92)	–	.09 (0.08–0.10)	8969.686	3782	−9784.488	74.971	157.488 (1)	0.00
E^b^	–	–	1.00	10732.090	3783	−8907.059	951.869	1919.892 (2)	0.00
FN 16 months									
Saturated				9666.282	3774	–9405.010			
ACE	.58 (0.50–0.67)	.22 (0.14–0.30)	.19 (0.17–0.22)	9674.930	3779	−9419.549	−14.449	8.648 (5)	0.124
CE^d^	–	.61 (0.58–0.64)	.39 (0.36–0.42)	9841.007	3780	−9340.283	79.266	166.077 (1)	0.00
AE^d^	.82 (0.79–0.84)	–	.18 (0.16–0.21)	9700.573	3780	−9410.500	5.4	25.643 (1)	0.00
E^b^	–	–	1.00	10730.845	3781	−8899.137	505.963	1051.915	0.00

−2LL, −2 log‐likelihood of data; *df*, degrees of freedom; BIC, Bayesian information criterion; A, additive genetic component of variance; C, shared environmental component of variance; E, unique environmental component of variance.

a All FF and FN scores were standardized for gestational age, age at questionnaire completion by the parents and sex. Standard ACE model‐fitting analyses for continuous data were used. The full ACE model was nested within the saturated model.

b Includes measurement error.

c Best‐fitting and most parsimonious model as specified by the lowest value of the BIC, indicating the solution which explains the observed variance and covariance with the fewest parameters.

d Submodels were nested within the full ACE model.

### Common genetic and environmental influences underlying both FF and FN

#### CT‐CT correlations

The CT‐CT correlations are shown in Table 3. The CT‐CT correlations were significantly higher for MZs (.67 and .64) than for DZs (.42 and .43), suggesting that genetic factors common to FF and FN were contributing to the phenotypic association between them.

**Table 3:** Cross‐twin cross‐trait correlations for FF and FN scores

CT‐CT ICCs (95% CI)	MZ	DZ
Twin 1 FF and Twin 2 FN	.67 (0.62–0.71)	.42 (0.38–0.47)
Twin 2 FF and Twin 1 FN	.64 (0.59–0.68)	.43 (0.39–0.48)

FF, food fussiness; FN, food neophobia; CT‐CT, cross‐twin cross‐trait correlations; ICCs, intraclass correlations; MZ, monozygotic; DZ, dizygotic.

#### Maximum likelihood structural equation modeling

**Figure 2:**
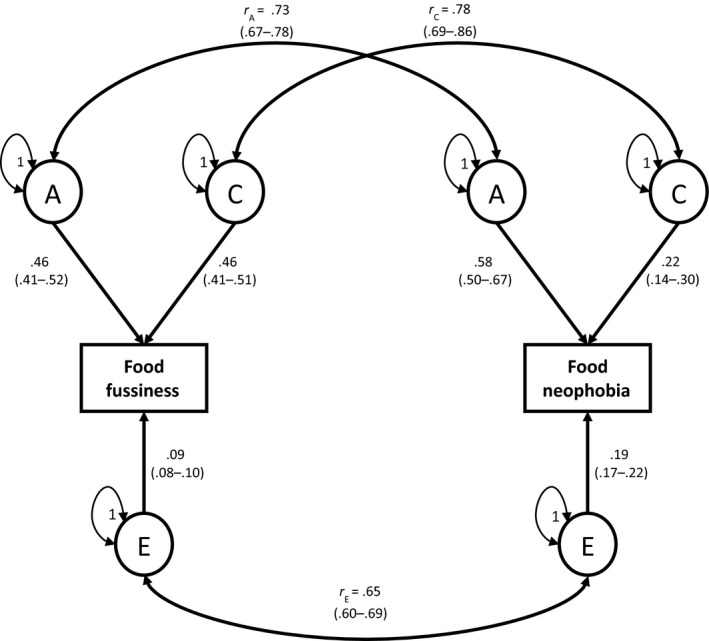
Full ACE bivariate correlated factors model for food fussiness (FF) and food neophobia (FN) at 16 months. The figure shows the full ACE correlated factors model showing the common etiology underlying ‘food fussiness’ and ‘food neophobia’. The rectangular boxes represent the measured phenotype (‘food fussiness’ and ‘food neophobia’). The circles indicate the latent factors of additive genetic effects (A), shared environmental effects (C), and nonshared environmental effects (E). The straight single‐headed arrows reflect standardized casual pathways with the variance explained by each latent factor (including 95% CIs). The curved, double‐headed arrows show the etiological correlations (genetic correlation, *r*_A_; shared environmental correlation, *r*_C_; and unique environmental correlation, *r*_E_); these indicate the proportion of genetic (*r*_A_), shared environmental (*r*_C_), and unique environmental (*r*_E_) influences that are common across the two phenotypes

The A, C, and E parameter estimates from the bivariate model were in keeping with those derived from the univariate analyses (Figure 2). The genetic, shared environmental, and unique environmental correlations (the ‘etiological correlations’) between FF and FN were all high (.65–.78) and significant, indicating that many of the same genetic and environmental influences underlie the two phenotypes. Nevertheless, the correlations were not complete (<1.0) suggesting some distinct influences also underlying each phenotype.

The bivariate estimates quantified the extent to which common genetic, shared environmental, and unique environmental factors underlying FF and FN explained the phenotypic association between them. Common additive genetic factors explained approximately half of the phenotypic association between FF and FN (bivariate A: .53; 95% CI: 0.46–0.62). Shared environmental factors common to both traits explained approximately one‐third of the phenotypic association (bivariate C: .35; 95% CI: 0.26–0.42). Unique environmental factors underlying both FF and FN were the least important in explaining the observed phenotypic association (bivariate E: .12; 95% CI: 0.10–0.14).

## Discussion

This is the first study to compare directly the etiology of FF and FN in the same sample. A key observation was that the shared environment played a significantly more important role in shaping FF (.46; 95% CI: 0.40–0.51) than FN (.22; 95% CI: 0.14–0.30). However, genetic influences were not significantly higher for FN (.58; 95% CI: 0.50–0.67) than for FF (.46; 95% CI: 0.41–0.52). The two phenotypes were highly correlated (.72), and shared many of the same underlying influences denoted by the high genetic (*r*_A_ = .76), shared environmental (*r*_C_ = .78), and unique environmental correlations (*r*_E_ = .65).

These findings suggest that, in line with previous research, the home and familial environment play a more important role in shaping FF than FN in early life. A key intervention to attenuate fussy eating behavior is repeated exposure to the problem food; the premise being that the more a child tries a food, the more familiar and the more acceptable it becomes (Fildes, van Jaarsveld, Wardle, & Cooke, 2014). This is an avenue through which parents might modify FF and FN, accounting for some of the observed shared environment effect for each trait. However, this strategy may fail more often with a highly neophobic child, perhaps explaining the lower impact of the shared environment for FN than for FF.

Both phenotypes showed some shared environmental influence suggesting that there is an opportunity for these traits to be modified through parental behavior. Coercive parental feeding styles have been associated with higher levels of fussy eating in children (Carruth & Skinner, 2013; Galloway, Fiorito, Lee, & Birch, 2005), while parental modeling of healthy eating behaviors has been linked to lower levels of FF and may therefore provide a strategy for parents to attenuate a child's food avoidant tendencies (Palfreyman, Haycraft, & Meyer, 2015).

The high genetic correlation (*r*_A_ = .73) derived from the bivariate analyses, suggests pleiotropic genetic effects underlying both behaviors. The comparably high shared and nonshared environmental correlations (*r*_C_ = .78 and *r*_E_ = .65) between FF and FN indicate that most of the environmental factors underlying these behaviors are also the same. In spite of the high etiological correlations for all of genetic, shared, and unique environmental influences, the bivariate estimates indicated that common genetic factors were particularly important in explaining the observed phenotypic correlation between FF and FN; common genetic influences explained over half of the phenotypic association (Biv A: .53; 95% CI: 0.46–0.62).

We also demonstrated that FN and FF are under moderate genetic control at this young age, fitting with anecdotal reports from parents that these traits emerge early, are somewhat innate, and difficult to modify (Galloway et al., 2003). The heritability estimates for FF and FN in this study are also in keeping with the magnitude of estimates observed for other appetitive characteristics in infancy and childhood. In Gemini, we previously reported high heritability estimates for appetitive traits in infancy (.53–.84; Llewellyn, van Jaarsveld, Johnson, Carnell, & Wardle, 2010); and for appetite in 10‐year‐old children (.62) from the Twins Early Development Study (TEDS; Llewellyn, van Jaarsveld, Boniface, Carnell, & Wardle, 2008). Food preferences at 3 years of age in Gemini (Fildes, van Jaarsveld, Llewellyn, et al., 2014) and 4 years of age in TEDS (Breen, Plomin, & Wardle, 2006) have also shown moderate heritability (.20–.78).

Interestingly, the heritability of FN was lower at this very young age than estimates reported in previous studies of children (Cooke et al., 2007; Faith et al., 2013) and adults (Knaapila et al., 2011). It is commonly observed that genetic expression increases across the life course; this has been reported for a variety of behavioral traits (Bergen, Gardner, & Kendler, 2007), and for adiposity itself (Llewellyn, Trzaskowski, Plomin, & Wardle, 2014). Likely explanations for developmental increases in heritability are both increasing active *and* evocative gene–environment correlations (GxE_R_). An ‘active’ GxE_R_ is the phenomenon whereby an individual increasingly selects out environments that reinforce their genetically determined trait (e.g. a fussy child avoiding foods they dislike); and an ‘evocative’ GxE_R_ refers to an individual increasingly eliciting certain environmental responses to their genetically determined trait (e.g. a parent giving a fussy child limited exposure to disliked foods). In contrast, passive GxE_R_ refers to the ‘double whammy’ of a child inheriting *both* genes *and* environment related to their parents’ and their own genetically determined trait (e.g. a child inheriting genes that predispose them to be fussy, as well as growing up with a fussy parent who creates an environment that nurtures fussiness). In infancy, passive GxE_R_ would be expected to be greatest, as the child is not yet independent and the parents largely control the eating environment. Gene–environment correlations increase the heritability of the trait because over time they strengthen similarities between individuals who are closely genetically related. We hypothesize that while passive GxE_R_ in relation to FF and FN will decrease with age, active and evocative GxE_R_ will increase. We therefore expect that heritability estimates for FF and FN will increase with development as children gain independence to ‘act out’ on their genetic propensities to be fussy, and increasingly elicit environmental responses to their fussiness.

### Implications

The first large study of children meeting the criteria for the new DSM‐5 diagnosis, ‘Avoidant/restrictive food intake disorder’ (ARFID; American Psychiatric Association, 2013), reported selective eating from early childhood to be the most typical characteristic (Fisher et al., 2014). Although the ARFID diagnosis is more sensitive compared to the DSM‐4 diagnosis of ‘Feeding disorder of infancy or early childhood’, the clinical presentations covered by this diagnosis and their underlying etiology may differ substantially (Zucker et al., 2015). Identifying common genetic and environmental contributions to FF and FN, two of the many conditions featuring food avoidance, strengthens ARFID as an all‐encompassing diagnosis of an underlying disorder. Categorizing restrictive eating behaviors into one overarching diagnosis may simplify referral to specialized treatment (Kreipe & Palomaki, 2012).

Demonstrating substantial shared genetic influence underlying both FF and FN indicates that genome‐wide association studies might be able to identify common genetic variants underlying both traits. Understanding the biological pathways through which common genes influence both traits will provide important insights that may advance our understanding of the mechanisms underlying these behaviors, opening up opportunities for the development of targeted behavioral interventions. Research has already shown that the association between higher FF and lower liking for fruit and vegetables is largely genetically mediated; that is, rejection of fruit and vegetables may partly result from a genetic predisposition toward FF more generally (Fildes, van Jaarsveld, Cooke, Wardle, & Llewellyn, 2016). Establishing the common genetic links between traits such as FF and FN contributes to our understanding of the etiology of a broad range of food avoidant eating behaviors.

The lower heritability of FF and FN at 16 months compared to higher estimates in older children (Cooke et al., 2007; Faith et al., 2013) points toward early childhood as a key time to intervene preemptively, as there may be more opportunity for environmental modification. Given the considerable influence of environmental factors on these behaviors, it would be useful for future research to identify specific modifiable environmental influences that shape FF and FN in early life so that they might be selectively targeted for interventions.

Mealtime conflicts are positively related to fussiness in children (Fulkerson, Story, Neumark‐Sztainer, & Rydell, 2008; Godfrey, Rhodes, & Hunt, 2013). Educating parents about the benefit of decreasing mealtime conflicts might provide an avenue for environmental modification to reduce FF and FN behavior (Bryant‐Waugh et al., 2010). At the same time, demonstrating a substantial genetic basis to both of these traits has the potential to divert blame away from parents as the main nurturers of these behaviors.

Repeated exposure and reward can be successful strategies for improving children's acceptance of disliked foods (e.g. vegetables; Corsini, Slater, Harrison, Cooke, & Cox, 2013). Given that our study supports a substantial shared etiology of FF and FN, applying such parent‐led eating behavior change programs to fussy or food neophobic young children is likely to be effective in decreasing their expression.

### Strengths and limitations

Food fussiness and FN were parent‐reported and it is possible that parents with thinner children assigned higher FF and FN scores as an explanation for poorer weight gain, and weight is highly heritable. However, the CEBQ is a reliable measure that has been validated against behavioral measures of eating behavior in the laboratory. Interpretation needs to consider that this division of the FF subscale of the CEBQ has face validity but has not been validated against the gold standard FN Scale (Pliner & Hobden, 1992). Direct comparison with previous research may thus be problematic. In addition, generalizability of the findings may be limited by the fact that twins are more likely to experience feeding difficulties, have lower birth weights, and are born more prematurely than singletons.

Heritability as assessed by any twin study is always only population and time‐specific. A strength of this study is the large sample size, permitting reliable parameter estimates to be established, with narrow 95% confidence intervals. Lastly, the bivariate design provides a unique insight into the relative importance of genetic or environmental influences on FF and FN at a key developmental stage.

## Conclusion

The findings from this study suggest there is significant genetic influence on FF and FN during early life. Shared environmental effects were found to explain a significantly greater proportion of the variation in FF than FN, suggesting that experiential factors in the home environment appear to be the most salient in explaining etiological differences in interindividual variation of FF compared to FN. Nevertheless, both traits largely share a common etiology.

**Key points**

- Food fussiness (FF) and food neophobia (FN) are restrictive eating phenotypes. Parents and clinicians consider these behaviors to be problematic because excessively fussy children may under eat or only accept a restricted number of foods.
- This twin study revealed the expression of FN and FF to be under moderate genetic control in early childhood.
- Largely shared environmental and genetic factors influenced variation in these behaviors, suggesting a common etiology of these traits.
- The considerable genetic influence on these tendencies in young children diverts the blame away from the home environment. The shared etiology of FF and FN behaviors indicates that parent‐led eating behavior change programs for fussy or food neophobic children may be effective in decreasing the expression of both.

## Supporting information


**Table S1.** Items on the CEBQ used to calculate Food Neophobia and Food Fussiness scores.
**Table S2.** Parameter estimates (95% confidence intervals) for A, C, and E for males and females considering qualitative and quantitative sex differences in food neophobia.
**Table S3.** Parameter estimates (95% confidence intervals) for A, C, and E for males and females considering qualitative and quantitative sex differences in food fussiness.

**Appendix S1.** STROBE statement – list of items that should be included in reports of cohort studies.
**Appendix S2.** Flow of families through the Gemini study between 2007 and 2011.
