# Supplementary material for: Food fussiness and food neophobia share a common etiology in early childhood
Source: J Child Psychol Psychiatry. 2016 Oct 14;58(2):189–96. doi: 10.1111/jcpp.12647 (PMC5298015; doi:10.1111/jcpp.12647)
Supplement: Supplementary file 1 — Table S1. Items on the CEBQ used to calculate Food Neophobia and Food Fussiness scores. Table S2. Parameter estimates (95% confidence intervals) for A, C, and E for males and females considering qualitative and quantitative sex differences in food neophobia. Table S3. Parameter estimates (95% confidence intervals) for A, C, and E for males and females considering qualitative and quantitative sex differences in food fussiness. [file JCPP-58-189-s001.docx]

**Supporting Information for *Food fussiness and Food Neophobia share a common etiology in early childhood* by Smith et al.**

Table S1. Items on the CEBQ used to calculate Food Neophobia and Food Fussiness scores

| Food Neophobia items | Food Fussiness items |
| --- | --- |
| My child refuses new foods at first  My child enjoys tasting new foods*  My child is interested in tasting food s/he hasn’t tasted before*  My child decides that s/he doesn’t like a food, even without tasting it | My child enjoys a wide variety of foods*  My child is difficult to please with meals  My child refuses to eat certain types of food† |

*Items were rated by parents on a 5-point Likert scale with answers ranging from “never” to “always”.*

*indicates reversed items

†additional FF item

**Table S2.** Parameters estimates (95% Confidence intervals) for A, C and E for males and females considering qualitative and quantitative sex differences in Food Neophobia

| **Model** | **Male** | | | **Female** | | |  |  |
| --- | --- | --- | --- | --- | --- | --- | --- | --- |
|  | **A_m_^1^** | **C_m_^1^** | **E_m_^1^** | **A_f_^1^** | **C_f_^1^** | **E_f_^1^** | **r_A_^1^** | **r_C_^1^** |
| **Full sex limitation (r_A_=free)** | 0.50  (0.36-0.65) | 0.28  (0.15-0.41) | 0.22  (0.18-0.26) | 0.64  (0.51-0.74) | 0.20  (0.10-0.32) | 0.16  (0.14-0.19) | 0.5  (0.39-0.50) | 1.00 |
| **Full sex limitation**  **(r_C_=free)** | 0.50  (0.36-0.65) | 0.28  (0.15-0.41) | 0.22  (0.18-0.26) | 0.64  (0.51-0.74) | 0.20  (0.10-0.32) | 0.16  (0.14-0.19) | 0.5 | 1.00  (0.88-1.00) |
| **Common effects model** | 0.50  (0.36-0.65) | 0.28  (0.15-0.41) | 0.22  (0.18-0.26) | 0.64  (0.52-0.74) | 0.20  (0.10-0.32) | 0.16  (0.14-0.19) | 0.5 | 1.00 |
|  | **A** | | **C** | | **E** | | **Scalar** | |
| **Scalar Model** | 0.58  (0.49-0.67) | | 0.23  (0.15-9.21) | | 0.19  (0.17-0.21) | | 0.94  (0.90-0.99) | |
|  | **A** | | **C** | | **E** | | **r_A_** | **r_C_** |
| **Null model (no sex differences)** | 0.58  (0.50-0.66) | | 0.23  (0.15-0.31) | | 0.19  (0.17-0.22) | | 0.5 | 1.00 |

^1^ Abbreviations: A: additive genetic component of variance; C: shared environmental component of variance; E: unique environmental component of variance; r_A_: genetic correlation, r_c_ : shared environmental correlation, r_E_ : non-shared environmental correlation.

**Table S3.** Parameters estimates (95% Confidence intervals) for A, C and E for males and females considering qualitative and quantitative sex differences in food fussiness

| **Model** | **Male** | | | **Female** | | |  |  |
| --- | --- | --- | --- | --- | --- | --- | --- | --- |
|  | **A_m_^1^** | **C_m_^1^** | **E_m_^1^** | **A_f_^1^** | **C_f_^1^** | **E_f_^1^** | **r_A_^1^** | **r_C_^1^** |
| **Full sex limitation (r_A_=free)** | 0.42  (0.32-0.51) | 0.42  (0.34-0.52) | 0.15  (0.13-0.18) | 0.39  (0.32-0.48) | 0.56  (0.47-0.63) | 0.05  (0.04-0.06) | 0.5  (0.38-0.5) | 1.00 |
| **Full sex limitation**  **(r_C_=free)** | 0.43  (0.32-0.51) | 0.42  (0.34-0.52) | 0.15  (0.13-0.18) | 0.39  (0.32-0.48) | 0.55  (0.47-0.63) | 0.05  (0.04-0.06) | 0.5 | 1.00  (0.97-1.00) |
| **Common effects model**  **(rA=0.5, rC=1)** | 0.43  (0.33-0.51) | 0.42  (0.34-0.51) | 0.15  (0.13-0.18) | 0.39  (0.32-0.48) | 0.56  (0.47-0.63) | 0.05  (0.04-0.06) | 0.5 | 1.00 |
|  | **A** | | **C** | | **E** | | **Scalar** | |
| **Scalar Model** | 0.43  (0.37-0.48) | | 0.48  (0.42-0.53) | | 0.09  (0.08-0.11) | | 0.92  (0.88-0.95) | |
|  | **A** | | **C** | | **E** | | **r_A_** | **r_C_** |
| **Null model (no sex differences)** | 0.42  (0.37-0.48) | | 0.48  (0.42-0.53) | | 0.10  (0.09-0.11) | | 0.5 | 1.00 |

^1^ Abbreviations: A: additive genetic component of variance; C: shared environmental component of variance; E: unique environmental component of variance; r_A_: genetic correlation, r_c_ : shared environmental correlation, r_E_ : non-shared environmental correlation.
